# MicroRNA‐708 modulates Hepatic Stellate Cells activation and enhances extracellular matrix accumulation via direct targeting TMEM88

**DOI:** 10.1111/jcmm.15119

**Published:** 2020-05-28

**Authors:** Tao Xu, Linxin Pan, Liangyun Li, Shuang Hu, Hong Zhou, Chenchen Yang, Junfa Yang, Haodong Li, Yuming Liu, Xiaoming Meng, Jun Li

**Affiliations:** ^1^ Anhui Provincial laboratory of inflammatory and immunity disease School of Pharmacy Anhui Institute of Innovative Drugs Anhui Medical University Hefei China; ^2^ The Key Laboratory of Anti‐Inflammatory and Immune medicines Ministry of Education Hefei China; ^3^ Institute for Liver Diseases of Anhui Medical University Hefei China; ^4^ The School of Life Science Anhui Medical University Hefei China; ^5^ Division of Life Sciences and Medicine Department of Pharmacy Anhui Provincial Cancer Hospital The First Affiliated Hospital of USTC University of Science and Technology of China Hefei China; ^6^ Affiliated Psychological Hospital of Anhui Medical University Anhui Medical University Hefei China; ^7^ Hefei Fourth People's Hospital Hefei China; ^8^ Institute of Clinical Pharmacology Anhui Medical University Hefei China

**Keywords:** liver fibrosis, LX‐2 cells, miR‐708, TMEM88

## Abstract

Transmembrane protein 88 (TMEM88) is a potential 2‐transmembrane‐type protein that interacts with the PDZ domain of Dishevelled‐1 (DVL‐1), a crucial component of Wnt signalling pathway through its C‐terminal Val‐Trp‐Val (VWV) motif in Xenopus embryo cells. Since the significant function of β‐catenin in liver fibrosis, it is urgent to study the TMEM88 mechanism in liver fibrosis. The current research was for evaluating the function of TMEM88 in the process of the liver fibrosis and clarifying the inherent mechanism. The study found that TMEM88 is decreased in human fibrotic liver tissues. Functionally, TMEM88 significantly reduced the expression levels of α‐smooth muscle actin (α‐SMA) and collagen type I (Col.I) and repressed extracellular matrix (ECM) accumulation by restoring the balance between matrix metalloproteinases (MMPs) and TIMPs (tissue inhibitor of metalloproteinases). TMEM88 inhibited HSCs proliferation and evaluated the apoptosis of activated LX‐2 cells by regulating Wnt3a, Wnt2b and β‐catenin of Wnt/β‐catenin signalling pathway. Moreover, we demonstrated that miR‐708 particularly targeted TMEM88 3′‐UTR regions and down‐regulated the expression level of TMEM88 in TGF‐β1‐stimulated LX‐2 cells. MiR‐708 promoted the generation of ECM and cell activation in activated LX‐2 cells. These results determined that miR‐708 could promote HSCs activation and enhance ECM accumulation via direct targeting TMEM88 by Wnt/β‐catenin signalling pathway. This will provide a potential target for future research in the process of liver fibrosis.

## 1 INTRODUCTION

Liver fibrosis, the ultimate common pathway for chronic or iterative liver damage, is characterized by the accumulation of extracellular matrix (ECM).^1^, ^2^ Hepatic stellate cells (HSCs) are the main cell in liver fibrosis.^3^ Of note, activated HSCs with transforming growth factor‐β1 (TGF‐β1) are important in the excessive production of ECM proteins, more importantly, alpha‐smooth muscle actin (α‐SMA) and type I collagen (Col.I) which are recognized as fibrosis markers and are participated in a series of fibrotic processes.^4^, ^5^, ^6^ In addition, there is increasing evidence that matrix metalloproteinases (MMPs)/metalloproteinase tissue inhibitors (TIMPs) systems control the relationship between ECM synthesis and degradation.^7^, ^8^ The up‐regulated expression level of TIMP1 and the suppressed expression level of MMP2 resulted in subsequent accumulation of ECM.^9^ Therefore, finding an intrinsic target for inhibiting ECM accumulation from HSCs will be a new direction for studying the progression of liver fibrosis.

Transmembrane protein 88 (TMEM88) is transmembrane protein found on the cell membrane via blocking Wnt/β‐catenin signalling pathway.^10^, ^11^ Moreover, growing evidence reported that Wnt/β‐catenin signalling pathway participates in liver fibrosis progression.^12^ TMEM88 could mediate inflammatory cytokines secretion by Wnt/β‐catenin signalling pathway.^13^ Furthermore, chronic liver inflammation could lead to liver fibrosis.^14^, ^15^ Hereof, it is central to investigate the function of TMEM88 in liver fibrosis progression. LX‐2 cells are stabilizing and infinite source of human HSCs, retaining the key features of activated HSCs.^16^ The results showed that TMEM88 may be related to HSCs activation and the generation of ECM and proliferation by regulating Wnt/β‐catenin signalling pathway in activated LX‐2 cells. Furthermore, the expression level of TMEM88 was subsequently examined both in the human fibrotic liver tissues and activated LX‐2 cells.

MicroRNAs, short 20‐22 nucleotides, are recognized to hold essential parts in liver fibrosis progression by binding to the 3′‐untranslated region (3′‐UTR) of the target mRNAs.^17^, ^18^, ^19^ Analysis of microRNAs expression has identified a group of dysregulated miRNAs in liver fibrosis.^20^, ^21^ According to the bioinformatics tools, Mirtarbase and Mirbase, the upstream gene of TMEM88 is miR‐708. The role of miR‐708 has been found in hepatocellular carcinoma (HCC),^22^, ^23^ the expression level of miR‐708 was lower in HCC.^24^ Moreover, a unique character of HCC is its close association with liver fibrosis. More than 80% of HCC develop in liver fibrosis.^25^ Therefore, we assumed that miR‐708 is a target for liver fibrosis. However, the underlying mechanism of how miR‐708 affects the characters of liver fibrosis remains unclear. MiR‐708 was supposed to regulate the cell activation in activated HSCs by targeting TMEM88. In addition, we found that miR‐708 dramatically enhanced the Wnt/β‐catenin signalling pathway in LX‐2 cells and thereby aggravated liver fibrosis both in vivo and in vitro.

## 2 MATERIALS AND METHODS

### 2.1 Materials and reagents

Dimethyl sulphoxide (DMSO) (D2650) was purchased from Sigma Chemical. DMEM High Sugar Medium was purchased from HyClone. Foetal bovine serum (FBS) and Opti‐MEM were purchased from Gibco. Lipofectamine™2000 and Trizol were purchased from Invitrogen. TMEM88, MMP2, TIMP1, α‐SMA, COI.1 and β‐actin primers were purchased from Gene Pharma. MiR‐708 mimics and MiR‐708 negative control (NC), miR‐708 inhibitor and NC were purchased from Gene Pharma. Primary Antibody Dilution Buffer, RIPA Lysis Buffer and PMSF were purchased from Beyotime. ECL‐chemiluminescent kit was purchased from ThermoFisher Scientific. Recombinant TGF‐β1 was purchased from Peprotech. TMEM88 (sc‐135525) monoclonal antibody was purchased from Santa Cruz Biotechnology. Mouse anti‐β actin (TA‐09) monoclonal antibody was purchased from ZSGB‐BIO. MMP2 and TIMP1 monoclonal antibody were purchased from Boster Bioss. α‐SMA and Col.I monoclonal antibody were purchased from Boster Bioss. Wnt3a and Wnt2b monoclonal antibody were purchased from Boster Bioss. Peroxidase‐conjugated rabbit anti‐goat IgG and peroxidase‐conjugated goat anti‐mouse IgG were purchased from ZSGB‐BIO. Transcriptor First Strand cDNA Synthesis Kit was purchased from TaKaRa. TB Green^®^ Premix Ex Taq™ II was purchased from TaKaRa. Human Interleukin 6 (IL‐6) ELISA KIT (CSB‐E04638h) and Human Tumor necrosis factor α (TNF‐α) ELISA KIT (CSB‐E04740h) were purchased from Cusabio. FITC Annexin V apoptosis detection kit I (401007) was purchased from BestBio. DAPI was purchased from Sigma. Cell‐Light EDU Apollo567 In Vitro Kit (100T) was purchased from RiboBio. Paraformaldehyde was purchased from Electron Microscopy Sciences. Dual‐luciferase reporter assay was purchased from Promega. pEGFP‐C2‐TMEM88 plasmid was constructed and stored in our laboratory (Anhui, China).^13^

### 2.2 Specimen collection

Normal liver tissues (the control group) and human fibrotic liver tissues were taken from patients undergoing partial hepatectomy who undergone liver biopsy for staging and grading of liver fibrosis in the First Affiliated Hospital of Anhui Medical University. The control group included patients with normal transaminase activity, no history of liver disease or alcohol abuse and no history of HBV, HCV or HIV infection. Liver tissues were used for haematoxylin and eosin (H&E) and Masson's trichrome (MTS) staining. The study is in line with the standards set by the Helsinki Declaration, approving by the Health Medical Research Ethics Committee of Anhui Medical University (20190246). All participants signed the patient's informed consent form. The characteristics of patients and health donors are shown in Table 1.

**Table 1:** Characteristics of the subjects enrolled in the study of liver tissue

Parameters	Healthy donor	Patients
Case, n	8	9
Age, n	56.25 (47‐62)	54 (37‐72)
Sex, n (%)		
Male	5 (75%)	8 (75%)
Female	3 (25%)	1 (25%)
HCC, n (%)		
With	–	0 (12.5%)
Without	–	9 (87.5%)
Serum ALT, U/L	15.5 (±3.64)	129 (±113.78)
Serum AST, U/L	17.0 (±1.85)	122.88 (±89.2)

### 2.3 Luciferase reporter assay

pHG‐MirTarget‐TMEM88‐3′UTR plasmid and mutant 3′UTR‐TMEM88 (pHG‐MirTarget‐TMEM88‐3U) were obtained from HonorGen Biotechnology. LX‐2 cells were transplanted into a 24‐well plate in advance. LX‐2 cells were transfected with plasmid by Lipofectamine™2000. More concretely, TMEM88‐3′UTR‐WT plasmid gene (200 ng), miR‐708 mimics or NC control (60 nmol) was chosen, the same experiment was performed in the control group of mutant 3′UTR‐TMEM88. 2.25 μL Lipofectamine™2000 and 100 μL Opti‐MEM were used to detect results. LX‐2 cells were harvested after 48 hours of transfection and then lysed. Dual‐luciferase reporter assay was used to detect the luciferase activities.

### 2.4 Cell culture

LX‐2 cells were donated by Scott L. Friedman and were cultured with DMEM supplemented with 10% FBS and 1% penicillin and streptomycin. Cells were cultured at 37°C in a humidified atmosphere of 5% CO_2_ and 95% air.

### 2.5 RNA interference analysis

The small interfering RNA of TMEM88 (TMEM88‐siRNA) was purchased from GenePharma Corporation. LX‐2 cells were inoculated in 6‐well plate with DMEM culture medium for 24 hours before operation. Then, LX‐2 cells were transfected with TMEM88‐siRNA and negative control siRNA (NC siRNA) for 6 hours via Lipofectamine™2000 in Opti‐MEM medium. Meanwhile, LX‐2 cells were transfected with miR‐708 mimics (miR‐708 inhibitor) or NC (60 nmol), respectively, for 6 hours via Lipofectamine™2000 in Opti‐MEM medium. The sequences are as following:

TMEM88‐siRNA: F: 5′‐GGUGGCUGCCUUCAAUCUUTT‐3′, R: 5′‐AAGAUUGAAGGCAGCCACCTT‐3′;
Negative Control: F: 5′‐UUCUCCGAACGUGUCACGUTT‐3′, R: 5′‐ACGUGACACGUUCGGAGAATT‐3′.
MiR‐708 mimics: F: 5′‐AAGGAGCUUACAAUCUAGCUGGG‐3′, R: 5′‐CAGCUAGAUUGUAAGCUCCUUUU‐3′.
MiR‐708 mimics NC: F: 5′‐UUCUCCGAACGUGUCACGUTT‐3′, R: 5′‐ACGUGACACGUUCGGAGAATT‐3′.
MiR‐708 inhibitor: 5′‐CCCAGCUAGAUUGUAAGCUCCUU‐3′.
MiR‐708 inhibitor NC: 5′‐CAGUACUUUUGUGUAGUACAA‐3′.

### 2.6 EDU DNA incorporation assay

Cell proliferation was detected by standard EDU DNA incorporation assay. Exponentially growing LX‐2 cells plated on 13 mm glass coverslips. Then, pEGFP‐C2‐TMEM88, TMEM88‐siRNA and NC were transfected into cells with Lipofectamine™2000, respectively. After culture for 24 hours, the cells were labelled with 50 μmol/L EDU for 2 hours, and then rinsed twice with ice‐cold phosphate‐buffered saline (PBS). After labelling, LX‐2 cells were fixed in 4% paraformaldehyde for 30 minutes and rinsed with 2 mg/mL glycine for 5 minutes, then permeabilized with 1% Triton X‐100 for 10 minutes, rinsed with PBS for 5 minutes. Apollo staining was performed for 30 minutes and then Hoechst staining for 30 minutes. The images were taken by fluorescence microscopy (Olympus).

### 2.7 Flow cytometry

Apoptosis of LX‐2 cell was analysed by FITC Annexin V apoptosis detection kit I. The cells were seeded in a 6‐well plate at a density of 1 × 10^5^ cells per well and transfected with TMEM88 (pEGFP‐C2‐TMEM88, TMEM88‐siRNA) for 24 hours at 37°C in a CO_2_ incubator. After a wash with cold PBS, LX‐2 cells were re‐suspended in 1 × binding buffer, then stained with 5 μL FITC Annexin V and 5 μL Propidium iodide (PI). The flow cytometer was used to detect the apoptosis.

### 2.8 Quantitative Real‐Time PCR

The cell culture medium was abandoned from the 6‐well plate and cleaned 3 times by PBS. LX‐2 cells were cytolysis to total RNA by TRIzol. Then, RNA was transcripted reverse to generate cDNA. Relative levels of specific mRNA were determined using the RTqPCR Detection System with TB Green^®^ supermix according to the manufacturer’s instructions. The β‐actin gene was used as an internal control for normalization. The primers used for PCR amplification are shown in Table 2.

**Table 2:** Primer sequences for quantitative real‐time reverse transcription polymerase chain reaction

Gene	Primer pair	
TMEM88	F:5′‐CCTACAGCCGAGCCCTTTAT‐3′	R:5′‐CCCAGACCTTTCCTGATGTG‐3′
α‐SMA	F:5′‐GCTATTCAGGCTGTGCTGTC‐3′	R:5′‐GGTAGTCGGTGAGATCTCGG‐3′
Col.I	F:5′‐TGCTGCCTTTTCTGTTCCTT‐3′	R:5′‐AGTGCCTCTTTGCTGCTTTC‐3′
MMP2	F:5′‐GTTGGCTGTGCAATACCTAAA‐3′	R:5′‐AAGGTGCTGGGTAGGGAAGT‐3′
TIMP1	F:5′‐ACTGCCTTATACCAGCGTTATG‐3′	R:5′‐GTGTAGATGAACCGGATGTCAG‐3′
β‐actin	F:5′‐GCCAACACAGTGCTGTCTGG‐3′	R:5′‐CTCAGGAGGAGCAATGATCTTG‐3′

### 2.9 Immunohistochemistry

Human fibrotic liver tissues and normal liver tissues were fixed in paraffin after fixation in 10% neutral paraformaldehyde and stained for routine histology. Liver sections were deparaffinized in xylene and rehydrated in decreasing concentrations of ethanol, and antigen retrieval was achieved by microwave treatment in citrated saline for 15 minutes. The sections were then treated with 0.3% hydrogen peroxide for 15 minutes to block endogenous peroxidase activity, further blocked with 2% bovine serum albumin (BSA), and then with TMEM88 (1:100) and α‐SMA (1:100). The primary antibody was incubated for 16 hours at 4°C. The expression of TMEM88 and α‐SMA was visualized by 3,3′‐diaminobenzidine tetrahydrochloride staining. The slides were counterstained with haematoxylin before dehydration and installation.

### 2.10 Double immunofluorescence staining

The liver tissues were permeabilized with 0.2% Triton X‐100 containing 1% BSA for 10 minutes, and blocked with 5% BSA for 1 hours at room temperature. To determine the co‐localization of TMEM88 and α‐SMA, FITC‐conjugated TMEM88 probes in combination with Cy3‐conjugated anti‐α‐SMA antibody (1:50) were used in the hybridization assays. The cells were mounted with SlowFade Gold antifade reagent with DAPI, and images were taken using fluorescence microscopy. TMEM88 was shown as green fluorescence and α‐SMA as red fluorescence.

### 2.11 Western blotting

LX‐2 cells (1 × 10^5^) were seeded in the 6‐well plate and cleaned three times via cold PBS. LX‐2 cells were cytolysis to total protein by protein lysate (RIPA lysate: PMSF = 100 μL) on ice for 30 minutes. The protein was extracted and subsequently was electrophoresed for 1 hour. After electrophoresis, the protein was blotted onto the PVDF membrane by electro transfer. After blocking the non‐specific protein binding by BSA, the PVDF membrane was incubated overnight in the diluted primary antibody, and the primary antibody dilution ratios were TMEM88 (1:400), MMP2 (1:1000), TIMP1 (1:1000), α‐SMA (1:1000) and Co1.I (1:1000), respectively. The protein was incubated for 1 hour with the second antibodies, and then ECL‐chemiluminescent kit was used for Western blotting detection. Quantitative densitometry of the immunoblot images was performed by Image J V1.8.0 software.

### 2.12 Statistical analysis

Statistical data analysis was performed by SPSS ver.18.0. The differences in groups were checked by one‐way ANOVA. The data were presented as the mean ± standard error at least three times experiments independently. If the *P* value < .05, the data were considered significant difference, and if the *P* value < .01, the data were considered strongly significant difference.

## 3 RESULTS

### 3.1 TMEM88 was decreased in human fibrotic liver tissues and TGF‐β1‐stimulated LX‐2 cells

**Figure 1:**
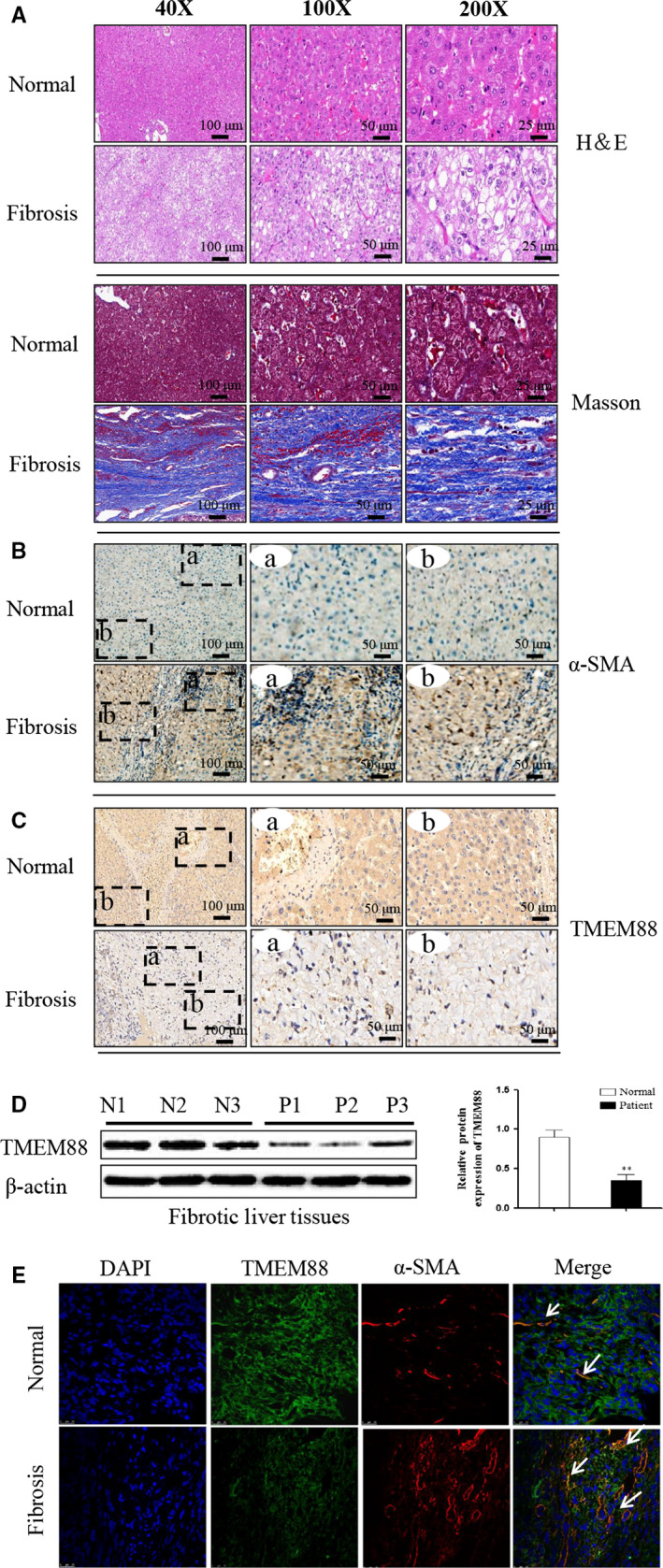
TMEM88 was decreased in human fibrotic liver tissues. A, The H&E and Masson stain in human fibrotic liver tissues and normal tissues. The results revealed that TMEM88 was significant decrease in human fibrotic liver tissues compared with normal group. The images were taken with 40‐fold, 100‐fold and 200‐fold magnification, respectively. The scale bars are shown as indicated. Pannoramic SCAN 150 (3DHISTECH, Budapest, Hungary) was used for the imaging. B, C, The immunohistochemistry of TMEM88, α‐SMA in human fibrotic liver tissues and normal tissues. The rectangular image in the left panel is magnified in the middle and right panels. The scale is shown in the figure. Pannoramic SCAN 150 (3DHISTECH, Budapest, Hungary) was used for the imaging. D, The protein expression level of TMEM88 was measured by Western blotting in human fibrotic liver tissues compared with normal group. E, ISH with anti‐TMEM88 probe and IHC with α‐SMA were performed to determine the co‐localization of TMEM88 (green) and α‐SMA (red) in human fibrotic liver tissues. Fluorescence microscope was used for the imaging (Olympus). Representative images from control and human fibrotic liver tissues are presented (×100) *P < .05 compared with the normal group

**Figure 2:**
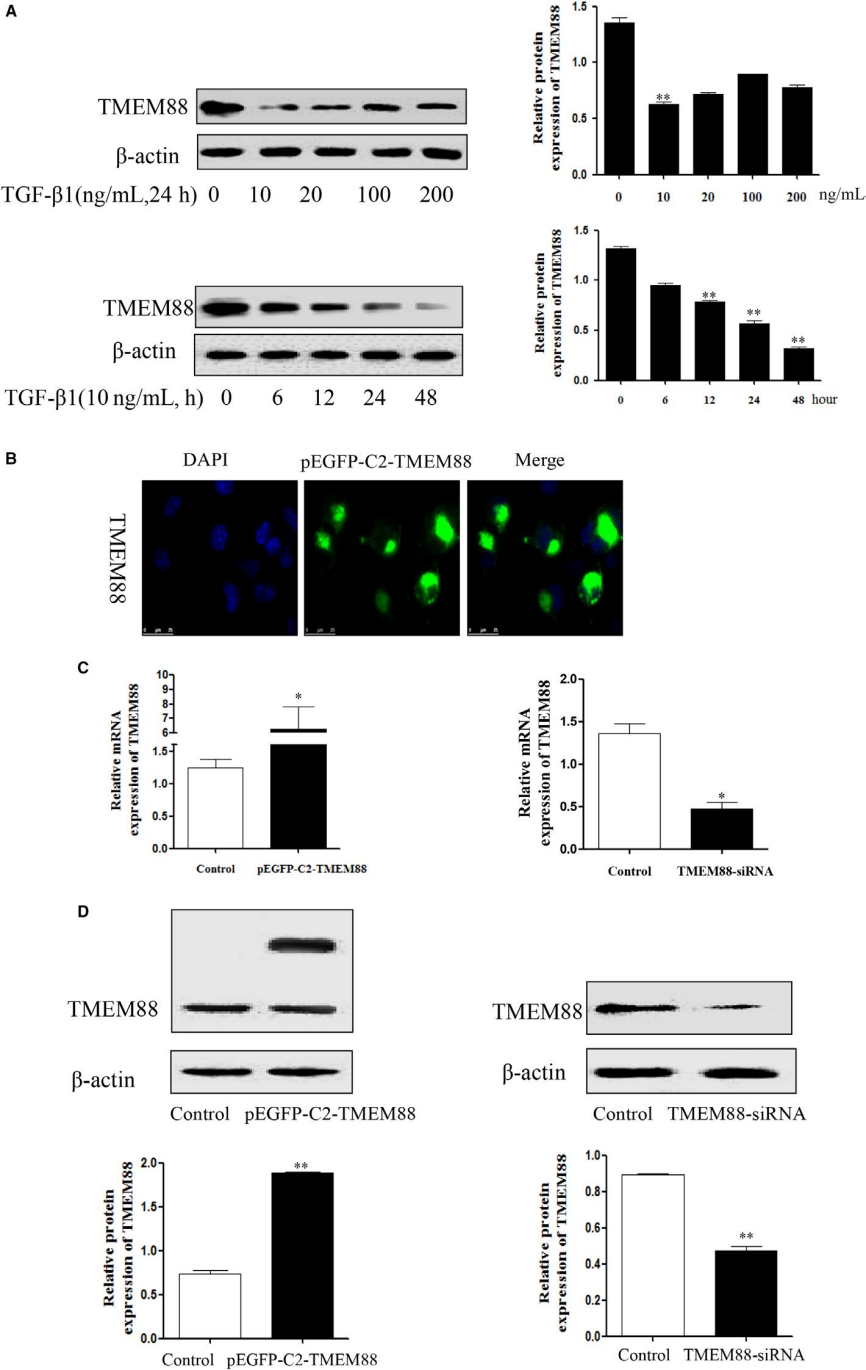
TMEM88 was decreased in TGF‐β1‐stimulated LX‐2 cells. A, The kinetic profiles of TMEM88 expression were observed in LX‐2 cells during activation induced by TGF‐β1 in different times and concentrations. B, The slides of LX‐2 cells transfected with pEGFP‐C2‐TMEM88 were taken out to immunofluorescence, and the result showed that the TMEM88 protein was mainly localized in cytoplasm. C, D, RT‐qPCR and Western blotting detected the expression level of TMEM88 both at mRNA and protein levels when transfected with pEGFP‐C2‐TMEM88 and pEGFP‐C2, respectively (GFP fluorescence protein‐positive cells were sorted after 48 h using MoFlo XDP cell Sorter of Center For Scientific Research, Beckman Coulter). The results were expressed as the mean ± standard of three different experiments. *P < .05 compared with the control group, ^#^ *P* < .05 compared with the control group

To determine whether TMEM88 was participated in liver fibrosis, the human fibrotic liver tissues were obtained for the study. First of all, the results of Masson staining and H&E staining displayed that human fibrotic liver tissues have severe liver steatosis, necrosis, regenerative nodules and fibrotic membrane formation compared with normal liver tissues (Figure 1A). Immunohistochemistry result demonstrated that the expression level of liver fibrosis marker (α‐SMA) was up‐regulated significantly compared with normal tissues (Figure 1B). Moreover, TMEM88 was detected in human fibrotic liver tissues. Indeed, immunohistochemistry and Western blotting result showed that the expression level of TMEM88 was down‐regulated in human fibrotic liver tissues compared with normal tissues (Figure 1C,D). Co‐labelling TMEM88 (ISH with anti‐TMEM88 probe) and α‐SMA (IHC) for co‐localization were used to detect the localization of TMEM88 in HSCs. Notably, the results of double immunofluorescence showed the co‐localization of TMEM88 with α‐SMA (Figure 1E). In order to further explore the change expression level of TMEM88 in TGF‐β1 stimulated LX‐2 cells. Moreover, the expression level of TMEM88 was observed in TGF‐β1‐stimulated LX‐2 cells at different times and concentrations. Western blotting analysis indicated the protein level of TMEM88 was decreased significantly at 24 hours in TGF‐β1‐stimulated LX‐2 cells (Figure 2A). Afterward, the expression level of TMEM88 was down‐regulated with increasing concentration of TGF‐β1, and the significantly protein levels of TMEM88 were picked at 10 ng/mL in TGF‐β1‐stimulated LX‐2 cells (Figure 2A). Based on these observations, we could conclude that the expression level of TMEM88 was inhibited in TGF‐β1‐stimulated LX‐2 cells.

### 3.2 TMEM88 inhibited cell activation in TGF‐β1‐stimulated LX‐2 cells

**Figure 3:**
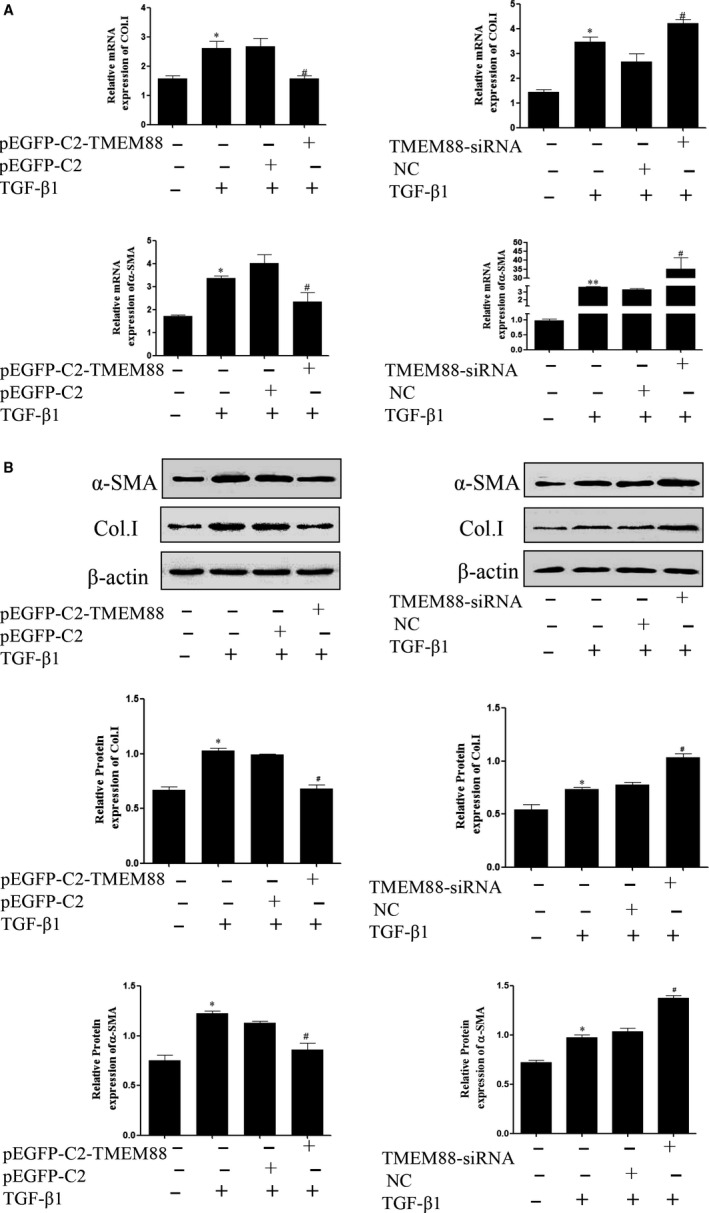
TMEM88 inhibited HSCs activation in TGF‐β1‐stimulated LX‐2 cells. A, The mRNA expression levels of α‐SMA and Co1.I were analysed with RT‐qPCR in activated LX‐2 cells transfected with pEGFP‐C2‐TMEM88 and TMEM88‐siRNA, respectively. The results showed that TMEM88 could inhibit the mRNA expression levels of α‐SMA and Co1.I. B, The protein expression levels of α‐SMA and Co1.I were measured by Western blotting analysis in activated LX‐2 cells transfected with pEGFP‐C2‐TMEM88 and TMEM88‐siRNA. The results showed that TMEM88 could inhibit the protein expression levels of α‐SMA and Co1.I. The results were expressed as the mean ± standard of three different experiments. *P < .05 compared with the normal group, ^#^ *P* < .05 compared with the control group

Furthermore, LX‐2 cells were respectively transfected with TMEM88‐siRNA and pEGFP‐C2‐TMEM88 to significantly decrease and increase the expression level of TMEM88 (Figure 2C,D). The result showed that pEGFP‐C2‐TMEM88 significantly inhibited the expression levels of α‐SMA and Col.I (Figure 3A,B). Apart from this, TMEM88 siRNA up‐regulated the expression levels of α‐SMA and Col.I (Figure 3A,B). In general, these results indicated that TMEM88 was correlated with HSCs activation and negatively regulated the expression levels of α‐SMA and Col.I.

### 3.3 TMEM88 alleviated MMPs/TIMPs system in TGF‐β1‐stimulated LX‐2 cells

**Figure 4:**
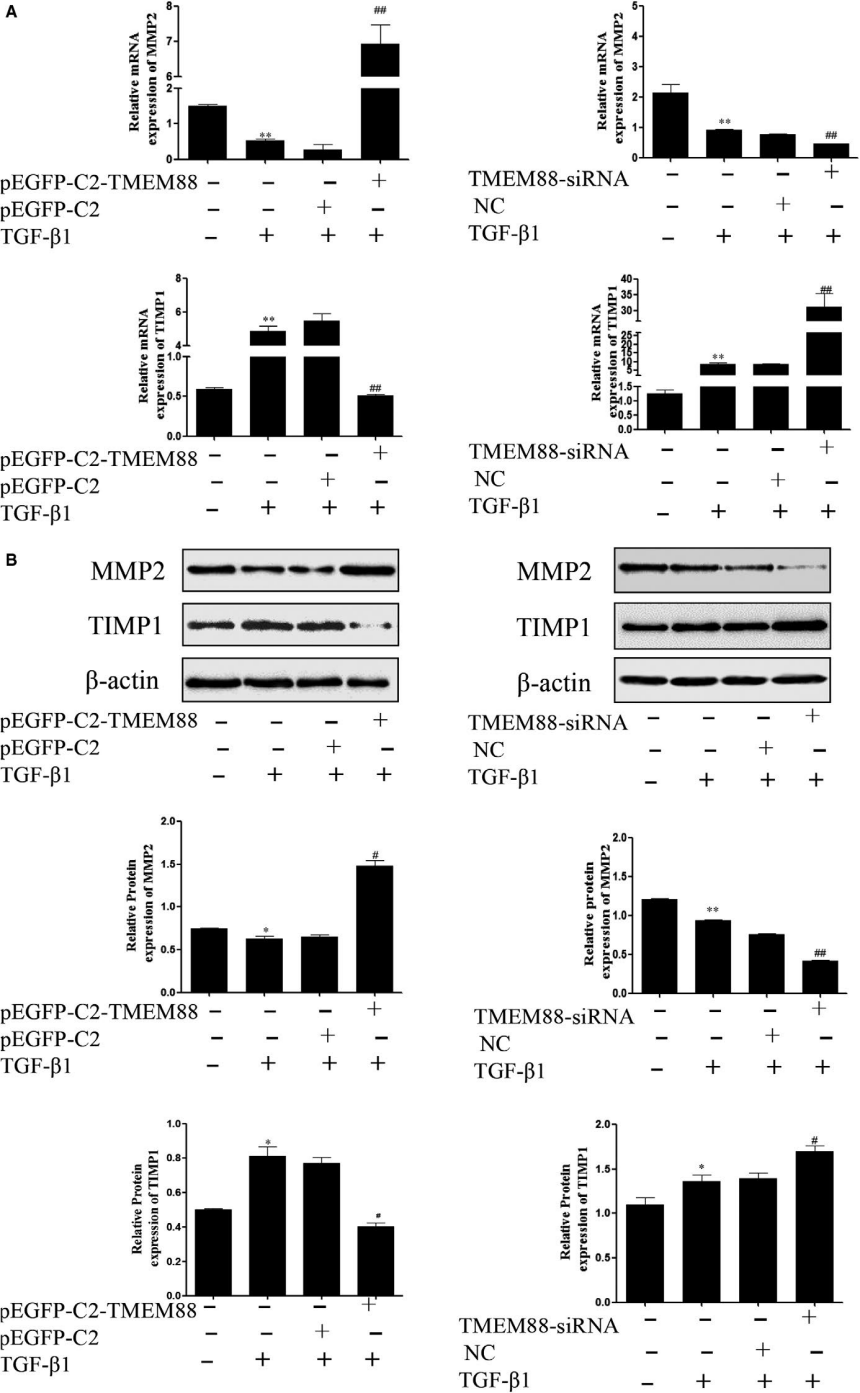
TMEM88 alleviated ECM accumulation in TGF‐β1‐stimulated LX‐2 cells. A, The mRNA expression levels of MMP2 and TIMP1 were analysed with RT‐qPCR in activated LX‐2 cells transfected with pEGFP‐C2‐TMEM88 and TMEM88‐siRNA, respectively. The results showed that TMEM88 could decrease the mRNA expression level of TIMP1, whereas increase the mRNA expression level of MMP2. B, The protein expression levels of MMP2 and TIMP1 were measured by Western blotting analysis in activated LX‐2 cells transfected with pEGFP‐C2‐TMEM88 and TMEM88‐siRNA. The results showed that TMEM88 could decrease the protein expression level of TIMP1, whereas increase the protein expression level of MMP2. The results were expressed as the mean ± standard of three different experiments. *P < .05 compared with the normal group, ^#^ *P* < .05 compared with the control group

To further observe the role of TMEM88 in the MMPs/TIMPs system, the mRNA and protein expression levels of MMP2 and TIMP1, key genes associated with the MMPs/TIMPs system, were detected in LX‐2 cells. RT‐qPCR assay showed that TMEM88 overexpression significantly decreased the mRNA expression level of TIMP1, whereas increased the mRNA expression level of MMP2 in TGF‐β1‐stimulated LX‐2 cells (Figure 4A). Moreover, the protein expression levels of MMP2 and TIMP1 were detected by Western blotting (Figure 4B), and the results were consistent with the mRNA expression. Additionally, TMEM88 silencing significantly up‐regulated the expression level of TIMP1, and markedly decreased the expression level of MMP2 in TGF‐β1‐stimulated LX‐2 cells (Figure 4A,B). Taken together, TMEM88 restored the balance in the MMPs/TIMPs system, thereby alleviated the ECM accumulation.

### 3.4 TMEM88 inhibited HSCs proliferation and promoted HSCs apoptosis in TGF‐β1‐stimulated LX‐2 cells

**Figure 5:**
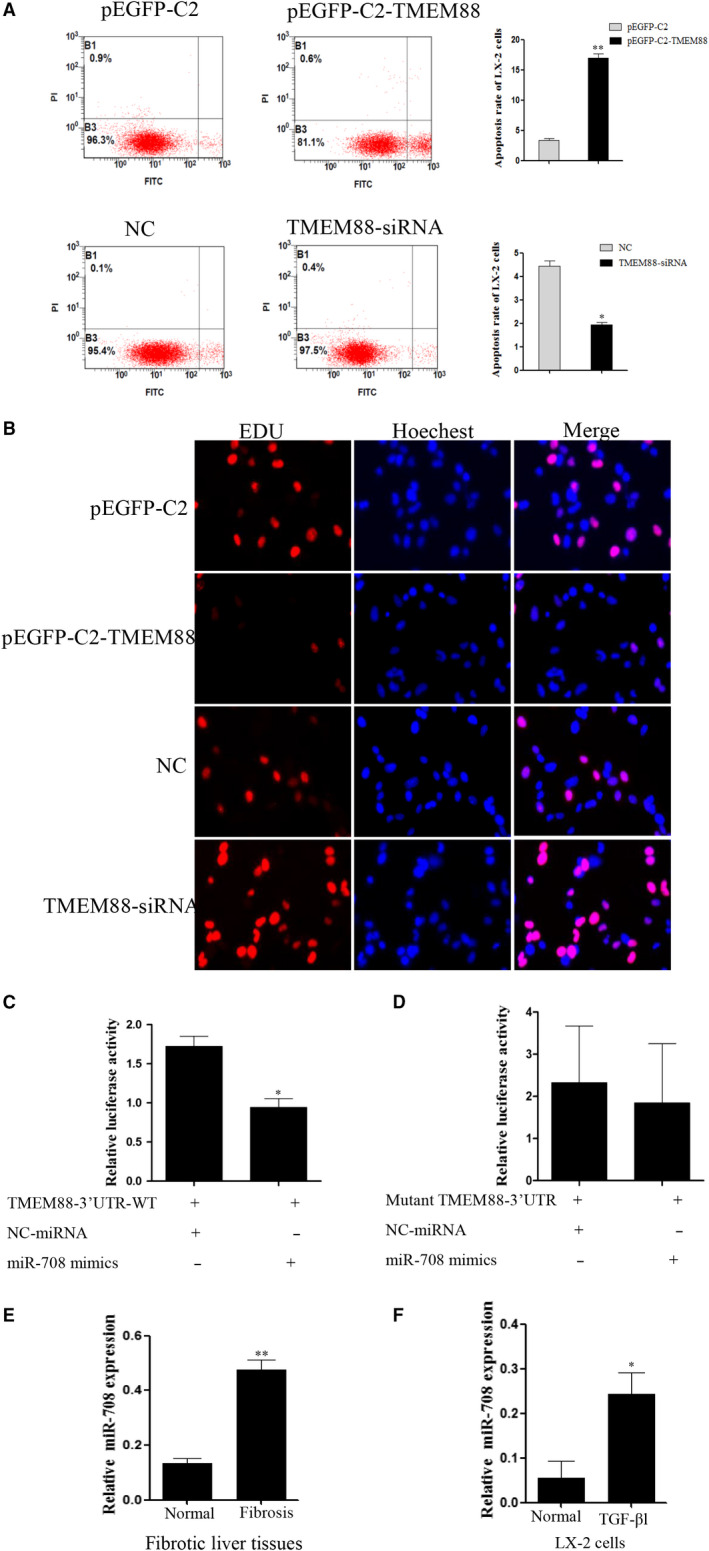
TMEM88 inhibited cell proliferation and promoted cell apoptosis in TGF‐β1‐stimulated LX‐2 cells. A, Cell apoptosis of LX‐2 cells was measured by flow cytometry analysis. B, Proliferation of LX‐2 cells was determined by EDU DNA incorporation assay. Fluorescence microscope was used for the imaging. C, The TMEM88‐3′‐UTR constructs or blank plasmid were transfected into LX‐2 cells with control or miR‐708 mimics, followed by dual‐luciferase assays. D, The mutant 3′UTR‐TMEM88 constructs or blank plasmid were transfected into LX‐2 cells with control or miR‐708 mimics, followed by dual‐luciferase assays. E, The mRNA expression level of miR‐708 was analysed with RT‐qPCR in human fibrotic liver tissues and normal tissues. F, The mRNA expression level of miR‐708 was analysed with RT‐qPCR in TGF‐β1‐stimulated LX‐2 cells. The results were expressed as the mean ± standard of three different experiments. *P < .05 compared with the normal group, ^#^ *P* < .05 compared with the control group

EDU DNA incorporation assay was used to detect the role of TMEM88 on cell proliferation in TGF‐β1‐induced LX‐2cells. Images of LX‐2 cells stained with EDU (red) to represent cell proliferation, whereas Hochest (blue) was used to label nucleus. The result showed that pEGFP‐C2‐TMEM88 or TMEM88 siRNA could lead to a noteworthy inhibition or promotion of cell proliferation in activated LX‐2 cells at 24 hours. The images demonstrated that pEGFP‐C2‐TMEM88 inhibited cell proliferation in activated LX‐2 cells, and the TMEM88 siRNA showed the opposite result (Figure 5B). Furthermore, the flow cytometry result indicated that TMEM88 siRNA significantly inhibited cell apoptosis in activated LX‐2 cells and the result of the TMEM88 overexpression showed the opposite effect (Figure 5A). Collectively, these results provided evidence that TMEM88 could promote cells apoptosis in TGF‐β1‐stimulated LX‐2 cells.

### 3.5 TMEM88 is a direct target of miR‐708 in LX‐2 cells

**Figure 6:**
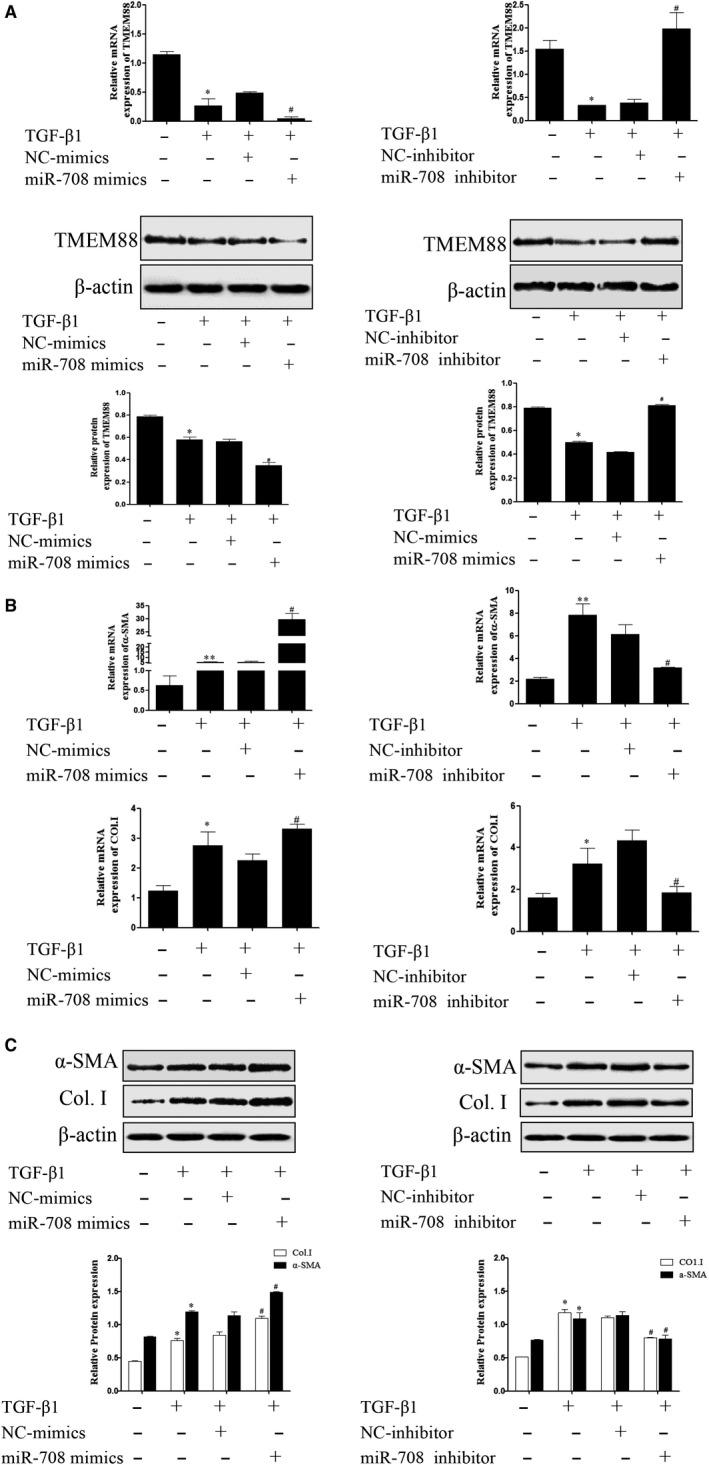
MiR‐708 promoted cell activation by targeting TMEM88 in TGF‐β1‐stimulated LX‐2 cells. A, The mRNA and protein expression level of TMEM88 was measured by Western blotting and RT‐qPCR analysis respectively in activated LX‐2 cells transfected with miR‐708 mimics and miR‐708 inhibitor, respectively. B, The mRNA expression levels of α‐SMA and Co1.I were analysed with RT‐qPCR in activated LX‐2 cells transfected with miR‐708 mimics and miR‐708 inhibitor, respectively. The results showed that miR‐708 could increase the mRNA expression levels of α‐SMA and Co1.I. C, The protein expression levels of α‐SMA and Co1.I were measured by Western blotting analysis in activated LX‐2 cells transfected with miR‐708 mimics and miR‐708 inhibitor, respectively. The results showed that miR‐708 could increase the protein expression levels of α‐SMA and Co1.I. The results were expressed as the mean ± standard of three different experiments. *P < .05 compared with the normal group, ^#^ *P* < .05 compared with the control group

Mirtarbase, a bioinformatics tool was used to predict target genes for miR‐708 to understand the underlying mechanisms by which miR‐708 regulates LX‐2 cells activation. The 3′‐UTR miRNA of TMEM88 contains putative miR‐708 binding sites predicted by prediction algorithm (Figure S1). To confirm miR‐708 regulates TMEM88 by binding to the corresponding 3′‐UTRs, the 3′‐UTR of TMEM88 was cloned from into the pmirGLO luciferase reporter vector. Then, the vectors of miR‐708 or control mimics were cotransfected with into LX‐2 cells. Double‐luciferase reporter assay was subsequently used to evaluate TMEM88 response to miR‐708. These funding revealed that miR‐708 significantly decreased TMEM88‐3′UTR‐WT luciferase activity in LX‐2 cells. Importantly, miR‐708 has no effect on mutant 3′UTR‐TMEM88 luciferase activity in LX‐2 cells (Figure 5C,D). RT‐qPCR result showed that the expression level of miR‐708 was up‐regulated in human fibrotic liver tissues compared with normal tissues, and the expression level of miR‐708 was up‐regulated in TGF‐β1‐stimulated LX‐2 cells (Figure 5E,F). Moreover, Western blotting result showed that mimics of miR‐708 down‐regulated the expression level of TMEM88 in LX‐2 cells. Conversely, miR‐708 inhibitor up‐regulated the protein level of TMEM88 in LX‐2 cells (Figure 6A). Herein, TMEM88 is a direct target of miR‐708 in LX‐2 cells.

### 3.6 MiR‐708 promoted cell activation by targeting TMEM88 in TGF‐β1‐stimulated LX‐2 cells

To determine the role of MiR‐708 on HSCs activation, the mRNA and protein expression levels of α‐SMA and Col.I which associated with cell activation were detected in TGF‐β1‐stimulated LX‐2 cells. Of note, miR‐708 mimics significantly increased the expression levels of α‐SMA and Col.I at mRNA and protein levels (Figure 6B,C). And results of RT‐qPCR and Western blotting analysis revealed that miR‐708 siRNA down‐regulated expression levels of α‐SMA and Col.I (Figure 6B,C). These results determined that miR‐708 promoted HSCs activation in TGF‐β1‐stimulated LX‐2 cells.

### 3.7 MiR‐708 treatment aggravated MMPs/TIMPs system by targeting TMEM88 in TGF‐β1‐stimulated LX‐2 cells

**Figure 7:**
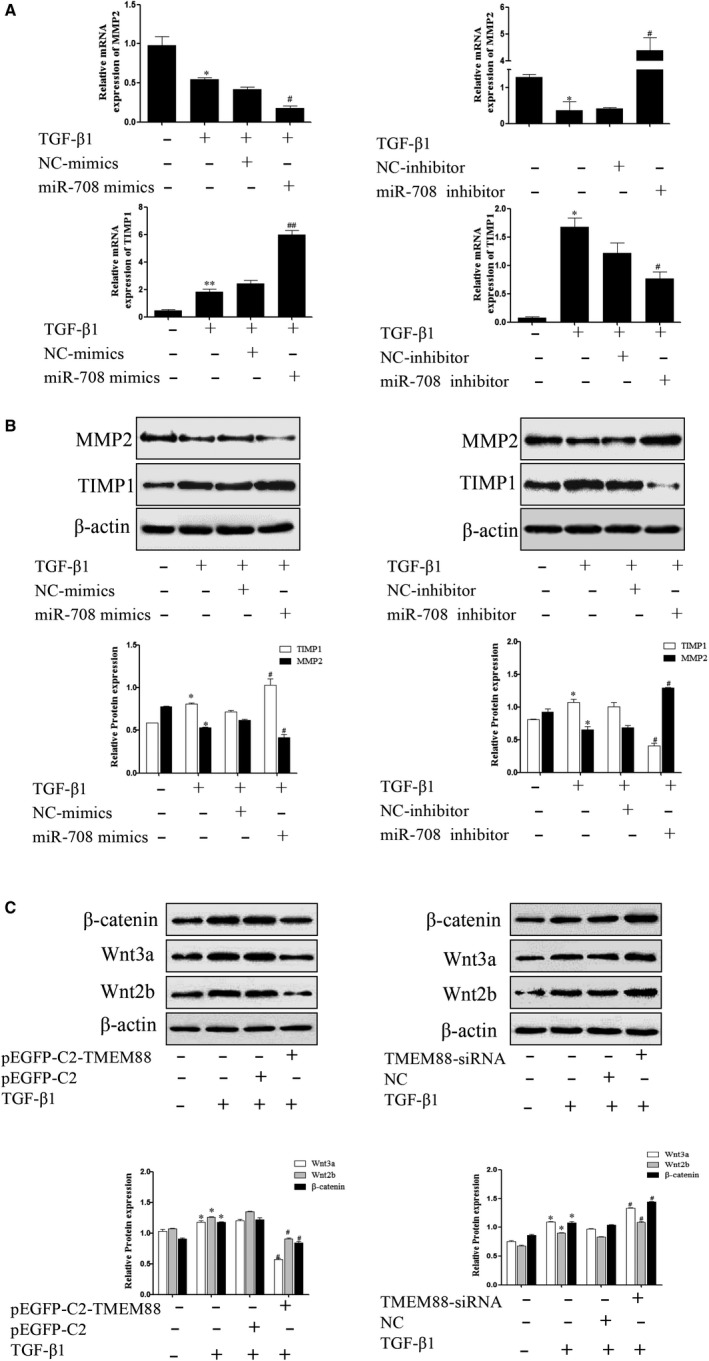
MiR‐708 enhanced ECM accumulation on Wnt/β‐catenin signalling pathway in TGF‐β1‐stimulated LX‐2 cells. A, The mRNA expression levels of MMP2 and TIMP1 were analysed with RT‐qPCR in activated LX‐2 cells transfected with miR‐708 mimics and miR‐708 inhibitor, respectively. The results showed that miR‐708 could increase the mRNA expression level of TIMP1, whereas decrease the mRNA expression level of MMP2. B, The protein expression levels of MMP2 and TIMP1 were measured by Western blotting analysis in activated LX‐2 cells transfected with miR‐708 mimics and miR‐708 inhibitor, respectively. The results were expressed as the mean ± SD of three different experiments. The results showed that miR‐708 could increase the protein expression level of TIMP1, whereas decrease the protein expression level of MMP2. C, The protein expression level of β‐catenin, Wnt3a and Wnt2b was performed in activated LX‐2 cells transfected with pEGFP‐C2‐TMEM88 and TMEM88‐siRNA, respectively. The results were expressed as the mean ± standard of three different experiments. *P < .05 compared with the control group, ^#^ *P* < .05 compared with the control group

To confirm the function of miR‐708 on MMPs/TIMPs system, miR‐708 mimics and miR‐708 inhibitor were respectively transfected in TGF‐β1‐stimulated LX‐2 cells. The results showed that miR‐708 mimics significantly decreased the protein and mRNA expression level of MMP2, whereas increased the protein and mRNA level of TIMP1 in TGF‐β1‐stimulated LX‐2 cells (Figure 7A,B). These results revealed that miR‐708 destroys the balance in the MMPs/TIMPs system by targeting TMEM88 in LX‐2 cells.

### 3.8 Effect of TMEM88 on Wnt/β‐catenin signalling pathway activity in TGF‐β1‐stimulated LX‐2 cells

Evidence indicated that the activation of Wnt/β‐catenin signalling pathway was tightly related to the HSCs activation and ECM accumulation. Mechanically, we examined the associated proteins of this signalling pathway. The results revealed that the expression levels of β‐catenin, Wnt3a and Wnt2b were significantly increased while transfected with TMEM88‐siRNA (Figure 7C). In addition, overexpression of TMEM88 promoted the protein expression levels of β‐catenin, Wnt3a and Wnt2b (Figure 7C). In summary, these results determined that TMEM88 might regulate the process of the liver fibrosis by Wnt/β‐catenin signalling pathway.

## 4 DISCUSSION

Excessive ECM accumulation will cause liver fibrosis which mainly generated by myofibroblasts in chronic liver disease,^26^, ^27^, ^28^ which could be regulated by Wnt/β‐catenin signalling pathway.^29^ β‐catenin is a key pro‐fibrosis and has been implicated in the pathogenesis of a variety of tissue fibrosis.^30^, ^31^ Meanwhile, Lee et al found that there are two isoforms of TMEM88: CRA‐a (17 kD), which inhibits Wnt/β‐catenin signalling pathway, and CRA‐b (25 kD), which is the motif that binds to the Dvl proteins and thus is not related to Wnt/β‐catenin signalling pathway.^32^, ^33^, ^34^, ^35^ Based on these observations, the role of TMEM88 in the progression of liver fibrosis remains unclear and needs further exploration. The purpose of this study is to find the character of TMEM88 in liver fibrosis with the secretion of ECM proteins and cell activation and to elucidate the mechanism underlying the correlation between TMEM88 and Wnt/β‐catenin signalling pathway in HSCs.

LX‐2 cells, a steady and stint‐less source of human HSCs, keep the pivotal features of stimulated HSCs, such as retinoid metabolism, and fibrogenesis.^36^ In this research, the expression level of TMEM88 was decreased obviously in LX‐2 cells stimulated by TGF‐β1. TGF‐β1, an important pro‐fibrosis factor and a major mediator in the pathogenesis of liver fibrosis, leads to the HSCs activation to aggravate the accumulation of ECM.^28^, ^37^ Additionally, TMEM88 was significantly decreased in human fibrotic liver tissues by Western blotting and immunohistochemistry. The expression levels of α‐SMA and Col.I are considered as a sign of HSCs activation. Notably, TMEM88 could inhibit the expression levels of α‐SMA and Col.I in TGF‐β1‐stimulated LX‐2 cells both at mRNA and protein levels, which indicated that TMEM88 was negatively regulated α‐SMA and Col.I to adjust HSCs activation. Emerging evidence indicated that MMP2 and TIMP1 are a major mediator in the development of ECM accumulation.^38^, ^39^ MMP2 and TIMP1, as representative cytokines to study the process of liver fibrosis, can participate in the progress of liver fibrosis.^40^ According to this study, TMEM88 could promote the expression level of MMP2 but inhibit the expression level of TIMP1 in LX‐2 cells. Conversely, knockdown of TMEM88 showed the opposite result. Based on the above results, we can conclude that TMEM88 plays a key role in regulating the accumulation of ECM in TGF‐β1‐stimulated LX‐2 cells. In terms of cell activity, cell proliferation is a method for determining cell viability as an important indicator of mechanisms of the involvement of certain genes, proteins and pathways in cell survival or death after exposure to toxic agents.^41^ EDU DNA incorporation assay was used to detect HSCs proliferation, which demonstrated that TMEM88 could lead to a significantly inhibition of proliferation in LX‐2 cells at 24 hours after transfected with pEGFP‐C2‐TMEM88. Furthermore, the flow cytometry was used to detect HSCs apoptosis, which indicated that TMEM88 could promote HSCs apoptosis in TGF‐β1‐stimulated LX‐2 cells compared with the control group.

Moreover, in the regulatory mechanism, activation of Wnt/β‐catenin signalling pathway is linked to liver fibrosis and TGF‐β1 is known as important stimuli of β‐catenin activity. Indeed, the present study found that TMEM88‐siRNA promoted the expression level of β‐catenin, Wnt3a and Wnt2b in TGF‐β1‐stimulated LX‐2 cells. On the contrary, overexpression of TMEM88 showed the opposite result. These results indicated that TMEM88 was a key part in aggravating the progression of the liver fibrosis by regulating Wnt/β‐catenin signalling pathway negatively.

It is a promising means of regulating liver fibrosis‐related pathways through miRNA therapy.^18^, ^42^ This study mainly found that miR‐708 is significantly elevated in TGF‐β1‐stimulated LX‐2 cells and human fibrotic liver tissues, while silencing of miR‐708 and overexpression of TMEM88 can inhibit HSC activation and reduce ECM accumulation. Bioinformatics tools (Mirtarbase and Mirbase) were used to predict the target genes of miR‐708. The 3′‐UTR of TMEM88 was cloned into the pmirGLO luciferase reporter vector and then cotransfected into LX‐2 with miR‐708 and miR‐708 NC respectively to determine if miR‐708 passed the corresponding 3′‐UTR combines to adjust TMEM88. The response of TMEM88 to miR‐708 was assessed by dual‐luciferase reporter assay. The study indicated that miR‐708 meaningfully inhibited TMEM88 luciferase activity in LX‐2 cells. In addition, double‐luciferase reporter assay clearly revealed that TMEM88 is a direct target of miR‐708 in HSCs. Functionally, the results showed that miR‐708 could regulate HSCs activation by increasing the expression levels of α‐SMA and Col.I. Meanwhile, miR‐708 enhanced the accumulation of ECM by decreasing the expression level of MMP2, whereas increasing the expression level of TIMP1 in TGF‐β1‐stimulated LX‐2 cells. Thence, we concluded that the miR‐708 could play an important role in liver fibrosis.

To summarize, our data indicated that TMEM88 is essential for the development of liver fibrosis and HSCs activation regulated by Wnt/β‐catenin signalling pathway in TGF‐β1‐stimulated LX‐2 cells. In future research, we will focus on the more features of TMEM88 in the development of liver fibrosis. Based on the function of TMEM88, research on targeted drugs of TMEM88 for liver disease will be subsequently carried out. Understanding the effects of TMEM88 on inflammation, autophagy, oxidative stress and other functions during liver fibrosis is a meaningful work, which can provide a bright future for the treatment of liver disease.

## CONFLICT OF INTEREST

The authors declare that they have no conflict of interest.

## AUTHOR CONTRIBUTIONS

Xiaoming Meng and Jun Li designed the study. Tao Xu and Linxin Pan participated in the collecting and analysing of the data. Liangyun Li and Zhou Hong finished the manuscript. Suwen Li, Shuang Hu and Chenchen Yang revised and edited the manuscript. Junfa Yang, Haodong Li and Yuming Liu reviewed the manuscript. All authors approved the final version of the manuscript for publication.

## Supporting information

Figure S1

## Data Availability

The authors confirm that the data supporting the findings of this study are available within the article.
