# Supplementary figures and images for: MicroRNA‐708 modulates Hepatic Stellate Cells activation and enhances extracellular matrix accumulation via direct targeting TMEM88
Source: J Cell Mol Med. 2020 May 28;24(13):7127–40. doi: 10.1111/jcmm.15119 (PMC7339227; doi:10.1111/jcmm.15119)

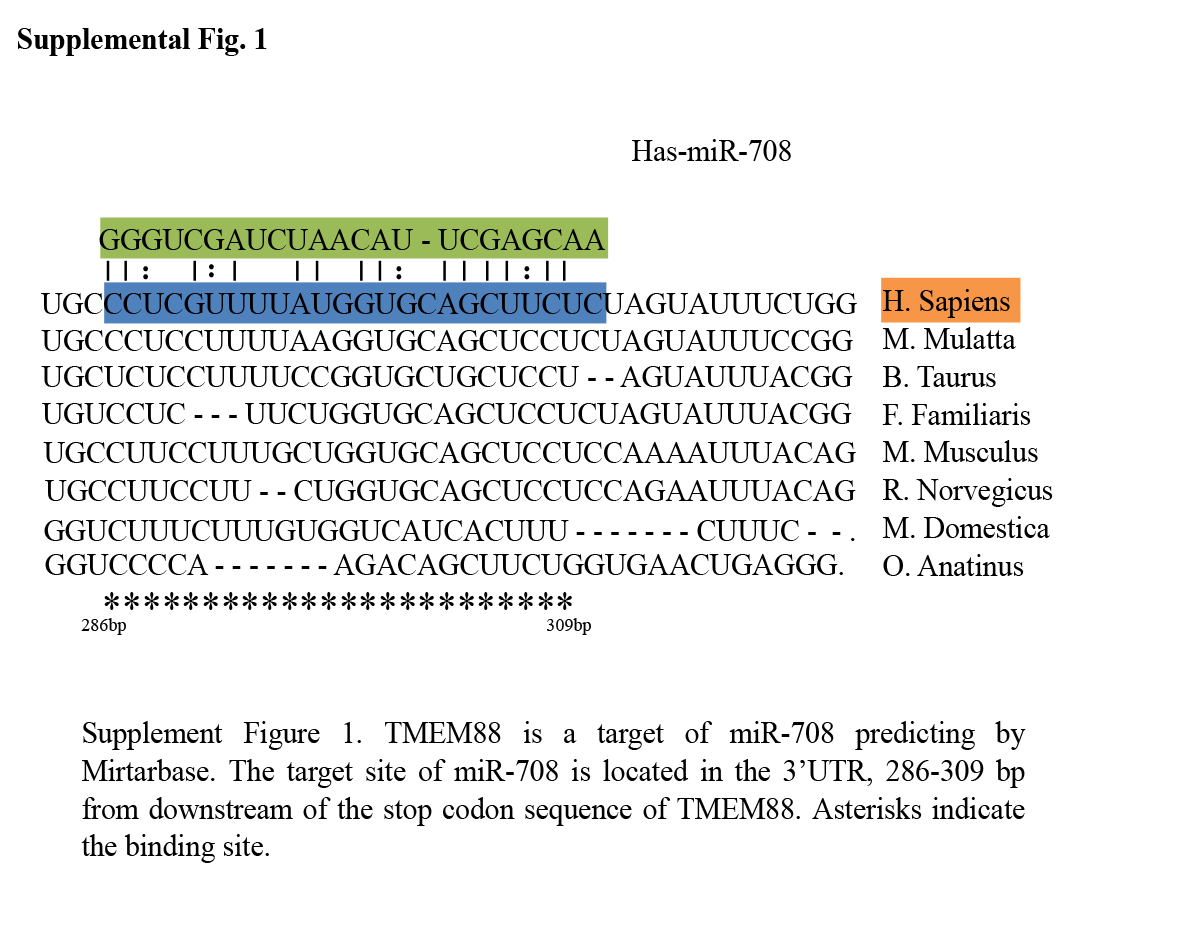

Supplement: Supplementary file 1 — Figure S1 [file JCMM-24-7127-s001.tif]
